# Gastrojejunostomy Closure Technique and Risk of Leak: an Evaluation in Ex Vivo Porcine Models

**DOI:** 10.1007/s11695-023-06470-0

**Published:** 2023-01-26

**Authors:** Bibek Das, Frances Ledesma, Hutan Ashrafian, Marcus Reddy, Omar A. Khan, Matyas Fehervari

**Affiliations:** 1 Department of Surgery and Cancer, Faculty of Medicine, Imperial College London, Commonwealth Building, Hammersmith Hospital Campus, Du Cane Road, London, W12 0NN UK; 2 Department of Surgery, Imperial College Healthcare NHS Foundation Trust, London, UK; 3 Department of Upper GI and Bariatric Surgery, St George’s Hospital NHS Foundation Trust, London, UK

**Keywords:** Gastric bypass, Bariatric surgery, Metabolic surgery, Gastrojejunostomy, Anastomotic leak

## Abstract

**Introduction:**

Roux-en-Y gastric bypass (RYGB) is one of the most commonly performed bariatric operations worldwide. Leaks following RYGB are rare, but the consequences can be devastating. Although most leaks occur at the gastrojejunostomy (GJ) anastomosis, there is a lack of data on modifiable technical factors that can reduce the risk of leaks. Therefore, we evaluated whether the leak pressure of a GJ linear stapled anastomosis is dependent on the closure technique.

**Methods:**

Two expert surgeons constructed gastric pouches and GJ anastomoses on ex vivo porcine models in a laparoscopic simulator using 30-mm and 45-mm endoscopic staplers. The GJ anastomosis was closed using either a single layer suture, double layer suture or stapler. The endpoints were leak pressure to air insufflation, measured by two independent observers, site of leak and internal circumference of the GJ anastomosis.

**Results:**

In total, 30 GJ anastomoses were constructed (30 mm, *n* = 15; 45 mm, *n* = 15). The GJ anastomosis was closed using single layer (*n* = 9), double layer (*n* = 9) and stapled techniques (*n* = 12). Inter-observer agreement was high. Stapled and double layer closures were more resilient than a single layer closure, with 75% (9/12) stapled closures remaining intact at < 70 mmHg. GJ stoma circumference was lower using a 30-mm stapler (64.8 mm vs 80.2 mm; *p* < 0.05) but independent of closure technique. The most common leak site was the corner of the closure (67%).

**Conclusion:**

In summary, the GJ anastomosis closure technique may be a modifiable factor to prevent anastomotic leak.

## Introduction

Roux-en-Y gastric bypass (RYGB) is one of the most common bariatric operations performed worldwide. Anastomotic leak (AL) following RYGB is a rare (1–2%) but life-threatening complication and the most common site is the gastrojejunostomy (GJ) [1, 2]. There is limited data on modifiable technical factors that can reduce the risk of AL and no high-quality evidence to support any intervention to reduce the incidence of AL [3]. Previous studies have also suggested that a larger GJ stoma size may adversely affect weight loss outcomes, although results have been conflicting [4–6]. The closure technique of the GJ anastomosis may affect both the resilience of the anastomosis and stoma size, but no study has directly evaluated the closure technique on RYGB outcomes, and national registries do not routinely record this information. Therefore, we conducted an exploratory study using ex vivo porcine models to determine whether clinically relevant biomechanical parameters (stoma size and burst pressure) are dependent on the closure technique.

## Methods

**Fig. 1:**
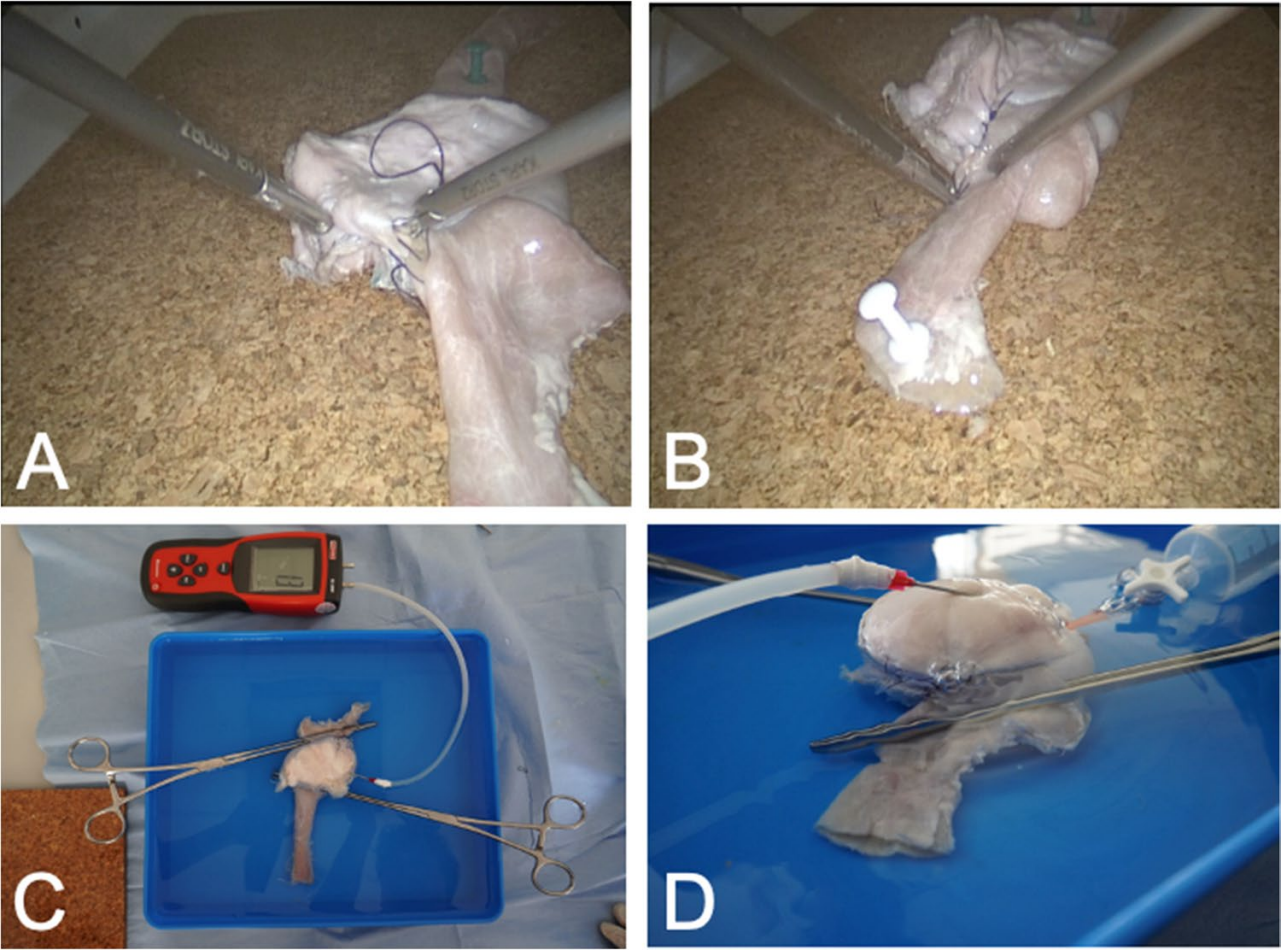
**A**–**B**, Laparoscopic suture closure of a porcine gastrojejunostomy model; **C**–**D**, demonstration of leak pressure testing

Using fresh porcine stomach and small bowel, two expert surgeons (having performed > 50 independent RYGB) constructed a GJ in a laparoscopic box simulator using a standardized study protocol, using a single firing of either a 30-mm or 45-mm linear stapler (Endo GIA™ with Tri-Staple™ Technology, Medtronic, Minnesota, USA). The opening in the GJ was closed using one of three methods: single layer suture (3–0 polyglactin 910 in a continuous fashion tied in the centre) (Fig. 1A, B), two-layer suture (3–0 polyglactin 910 in a continuous fashion for the inner layer and an interrupted 2–0 silk suture for the outer layer) and stapled (using the same stapler reload used to construct the GJ anastomosis).

After completion, the specimen was removed and submerged in water for leak testing by two independent observers. Atraumatic bowel clamps were applied at the gastro-esophageal junction and 1 cm distal to the GJ anastomosis (Fig. 1C). Air was slowly insufflated into the gastric pouch using a syringe, needle and a three-way tap, while the internal pouch pressure was monitored using a calibrated differential pressure manometer. The leak pressure was defined as the pressure at which air bubbles were visible at the anastomosis (Fig. 1D). The pressure was not increased above 70 mmHg as significant resistance precluded further air insufflation. As a reference, the median intra-gastric pressure during flexible gastroscopy is 10 mmHg (3–17.8 mmHg) [7]. After leak testing, the pouch was opened and the maximum internal circumference of the GJ was measured using a suture with the stoma held opened at four points using hemostats. Inter-rater agreement for leak pressure and internal circumference measurements was high (intra-class correlation coefficient 0.98 and 0.95, respectively).

## Results

**Fig. 2:**
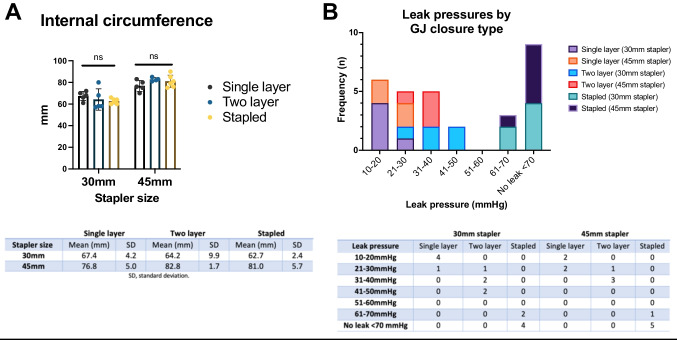
**A**, Maximum internal circumference of GJ by closure type; **B**, leak pressures by GJ closure type

In total, 30 GJ were constructed using a 30-mm stapler (*n* = 15) or a 45-mm stapler (*n* = 15) and closed using a single layer (*n* = 9), two layer (*n* = 9) or stapled technique (*n* = 12). The GJ stoma internal circumference was lower using a 30-mm stapler compared to a 45-mm stapler (64.8 mm vs. 80.2 mm; *p* < 0.0001), but this was independent of closure type (*p* = 0.78) (Fig. 2A).

Leak pressure testing revealed that two-layer suture closures were more resilient than a single layer closure, displaying higher leak pressures with both 30 mm (39.6 mmHg vs. 19.8 mmHg; *p* = 0.0012) and 45 mm (33.8 mmHg vs. 17.0 mmHg; *p* = 0.0018) stapled anastomoses (Fig. 2B). Stapled closures were also significantly more resilient than suture closures overall, with most resisting leaks at pressures < 70 mmHg (9/12 vs. 0/18; *p* = 0.001). All leaks occurred at the GJ closure site. The most common site of leak overall was the corner of the closure site (67%; *n* = 14/21), although leaks at the centre of the closure were more common after a single layer closure than a two-layer closure (6/9 vs. 1/8; *p* = 0.04).

## Conclusion

This study has evaluated the role of the GJ closure technique on stoma size and the risk of AL. The circumference of the GJ stoma was unaffected by closure type, suggesting that closure technique may have minimal effect on restriction and weight loss outcomes. We also found that stapled/two-layer closures were more resilient than single-layer closures, suggesting that these techniques may reduce the risk of AL. The recorded pressures suggest that careful endoscopy may be safely performed even in the early postoperative stage. Limitations of this study are the use of supra-physiological luminal pressures for leak testing and ex vivo porcine models which may have less resilience than perfused human tissue and cannot assess ischemic leaks or long-term sequelae such as strictures.

In conclusion, the GJ closure technique may be a modifiable factor to prevent AL. We suggest that national registries of RYGB should recommend recording the GJ closure technique routinely to determine the impact on surgical outcomes.

## Data Availability

All data generated or analysed during this study are included in this published article.
